# RAGE-aptamer attenuates deoxycorticosterone acetate/salt-induced renal injury in mice

**DOI:** 10.1038/s41598-018-21176-5

**Published:** 2018-02-08

**Authors:** Kensei Taguchi, Sho-ichi Yamagishi, Miyuki Yokoro, Sakuya Ito, Goh Kodama, Yusuke Kaida, Yosuke Nakayama, Ryotaro Ando, Nana Yamada-Obara, Katsuhiko Asanuma, Takanori Matsui, Yuichiro Higashimoto, Craig R. Brooks, Seiji Ueda, Seiya Okuda, Kei Fukami

**Affiliations:** 1 Division of Nephrology, Department of Medicine, Kurume University School of Medicine, Kurume, Japan; 2 Department of Pathophysiology and Therapeutics of Diabetic Vascular Complications, Kurume University School of Medicine, Kurume, Japan; 3 Department of Food Sciences and Nutrition, School of Human Environmental Sciences, Mukogawa Women’s University, Nishinomiya, Japan; 4 Department of Nephrology, Chiba University Graduate School of Medicine, Chiba, Japan; 5 Department of Medical Biochemistry, Kurume University School of Medicine, Kurume, Japan; 6 Division of Nephrology, Department of Medicine, Vanderbilt University Medical Center, Nashville, Tennessee USA; 7 Division of Nephrology, Department of Internal Medicine, Juntendo University, Tokyo, Japan

## Abstract

The mineralocorticoid receptor (MR) and its downstream signaling play an important role in hypertensive renal injury. The interaction of advanced glycation end products (AGE) with their receptor (RAGE) is involved in the progression of renal disease. However, the pathological crosstalk between AGE–RAGE axis and MR system in kidney derangement remains unclear. We screened DNA-aptamer directed against RAGE (RAGE-apt) *in vitro* and examined its effects on renal injury in uninephrectomized deoxycorticosterone acetate (DOCA)/salt-induced hypertensive mice. RAGE, GTP-bound Rac-1 (Rac1), and MR were co-localized in the podocytes of DOCA mice. The deletion of RAGE gene significantly inhibited mesangial matrix expansion and tubulointerstitial fibrosis in DOCA mice, which was associated with the reduction of glomerular oxidative stress, MR, Rac1, and urinary albumin excretion (UAE) levels. RAGE-apt attenuated the increase in carboxymethyllysine (CML), RAGE, nitrotyrosine, Rac1, and MR levels in the kidneys and reduced UAE in DOCA mice. Aldosterone (Aldo) increased nitrotyrosine, CML, and RAGE gene expression in murine podocytes, whereas CML stimulated MR and Rac1 levels, which were blocked by RAGE-apt. The present study indicates the crosstalk between the AGE–RAGE axis and Aldo–MR system, suggesting that RAGE-apt may be a novel therapeutic tool for the treatment of MR-associated renal diseases.

## Introduction

Hypertensive nephropathy (HN) is one of the common causes of end-stage renal disease requiring renal replacement therapy in industrialized countries^1,2^. Chronic hypertension induces arteriosclerosis in the renal afferent and efferent arterioles and subsequently causes ischemic changes in the glomeruli via the intrarenal renin–angiotensin–aldosterone (Aldo) system (RAAS) activation, thereby leading to renal damage, including podocyte loss, glomerular sclerosis, and tubulointerstitial fibrosis^3–5^. Furthermore, mineralocorticoid receptor (MR) activation in podocyte has been reported to accelerate the development and progression of HN^6^. Since the inhibition of MR activation attenuates Aldo-induced podocyte injury in an animal model of salt-sensitive HN^7^, the blockade of MR activation may be a promising therapeutic strategy for podocyte damage in HN. Indeed, several blood-lowering agents, such as MR antagonists and/or angiotensin II type 1 receptor blockers, show beneficial effects on HN^8,9^. However, the effects of these agents on HN are modest and partial; hence, pathways other than RAAS may also be involved in the progression of HN^10^.

Advanced glycation end products (AGE) are a heterogeneous group of molecules formed by a non-enzymatic reaction between reducing sugars and amino acids^11^. AGE modification of proteins causes not only structural alteration but also interacts with the receptor for AGE (RAGE), contributing to the development of numerous age-related devastating disorders, such as chronic kidney disease, cardiovascular diseases, and cancer^12–14^. Furthermore, since AGE activate Aldo–MR pathway through the interaction with RAGE on MRC-5 fibroblasts^15^, it is conceivable that the AGE–RAGE system may accelerate MR downstream pathways, thereby showing involvement in renal injury.

Aptamers are short, single-stranded DNA or RNA molecules that can bind with high affinity and specificity to a wide range of target proteins^16^. Recently, we reported that DNA-aptamer (DNA-apt) directed against AGE inhibited the binding of AGE to RAGE and attenuated renal injury in obese type 2 diabetic mice^17,18^. These findings suggest that aptamers may be a therapeutic tool in the prevention of AGE–RAGE-related disorders. Therefore, in this study, we screened a high-affinity DNA-apt directed against RAGE (RAGE-apt) using a combinatorial chemistry *in vitro*, and we examined its effects on renal injury in uninephrectomized deoxycorticosterone acetate (DOCA)/salt-induced hypertensive mice (DOCA mice). Furthermore, we explored how the AGE–RAGE axis activated MR downstream pathways using immortalized murine podocyte cells (MPC).

## Results

### Distribution of RAGE, GTP-Bound Rac1, and MR in the Glomeruli of DOCA Mice

**Figure 1:**
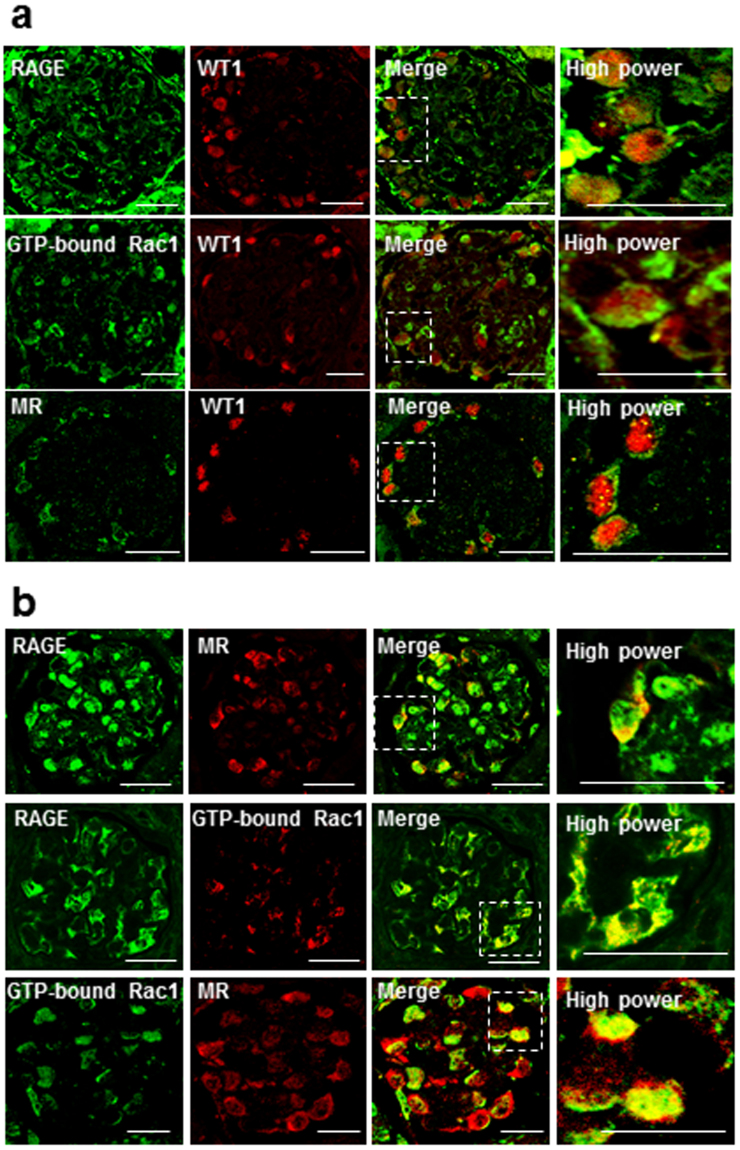
RAGE, GTP-bound Rac1, and MR were co-localized in podocytes of DOCA/salt-induced hypertensive mice. (**a**) Sections of kidney tissue from DOCA mice showing glomeruli co-stained for RAGE, GTP-bound Rac1, and MR (green), the podocyte marker WT1 (red), and overlaid images (yellow). (**b**) Sections of kidney tissue from DOCA mice co-stained for RAGE, GTP-bound Rac1, and MR and their merged images. RAGE, GTP-bound Rac1, and MR were together co-localized in podocytes from DOCA mice (arrows). All sections are 4-μm thin and evaluated by confocal microscopy. Bars = 20 μm. RAGE, receptor for advanced glycation end products; WT1, Wilms’ tumor 1; MR, mineralocorticoid receptor.

First, we investigated the co-localization and distribution of RAGE, MR, and Rac1 in the glomeruli of uninephrectomized DOCA/salt-induced hypertensive mice (DOCA mice) by an immunofluorescence double staining with RAGE, MR, GTP-bound Rac1, and Wilms’ tumor 1 (WT-1), a marker of podocytes. RAGE, GTP-bound Rac1, and MR were merged with WT-1 (Fig. 1a). Further, immunofluorescence double staining demonstrated that RAGE, GTP-bound Rac1, and MR were co-localized with each other (Fig. 1b) in podocytes in the kidneys of DOCA mice.

### Renal RAGE Expression in DOCA Mice

**Figure 2:**
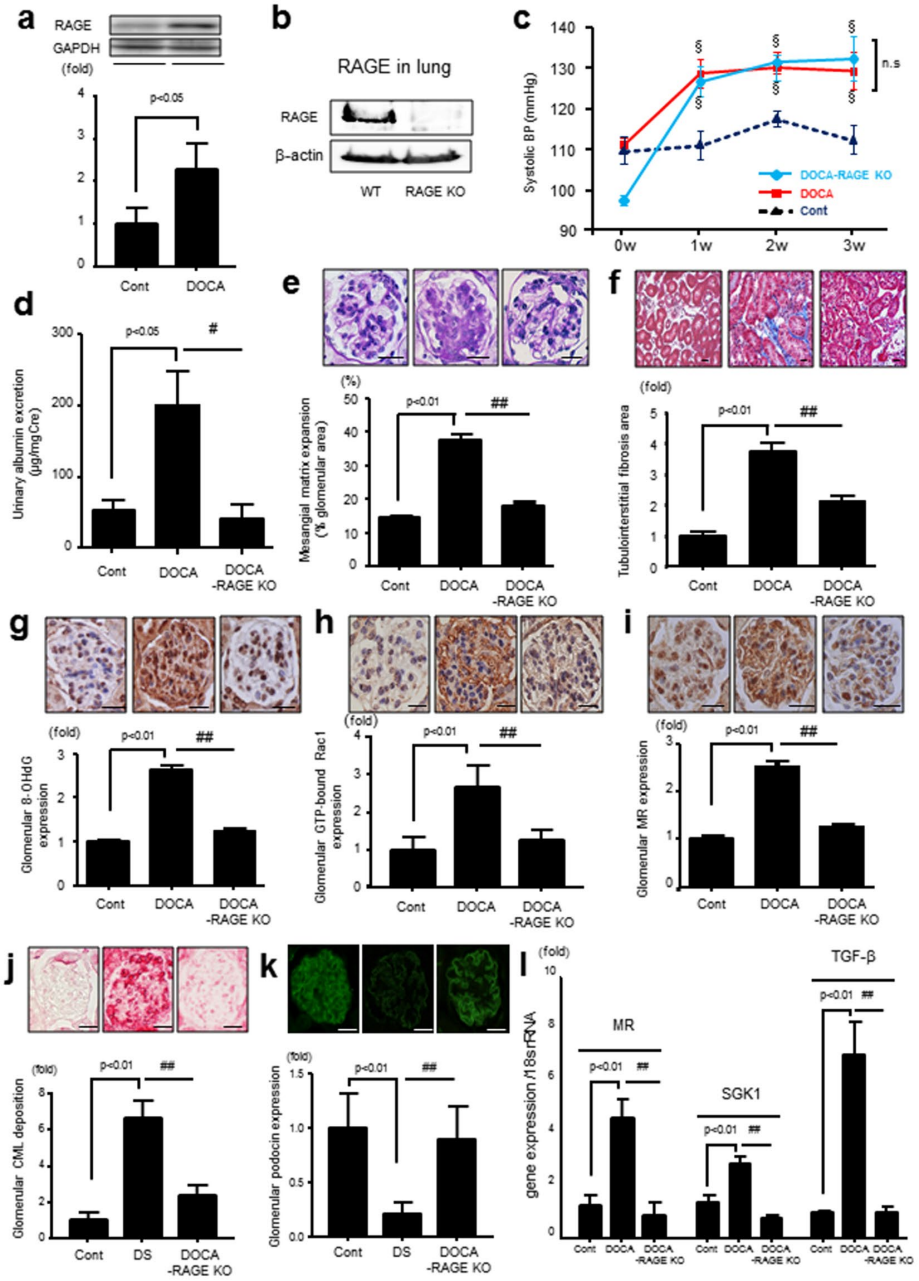
(**a**) RAGE protein expression in the kidney of Cont and DOCA mice (n = 3, respectively), (**b**) RAGE protein expression in lung of WT and RAGE-KO mice, (**c**) SBP levels of Cont, DOCA, and DOCA–RAGE-KO mice (n = 5–8 per group), ^§^p < 0.05 vs Cont, (**d**) UAE levels of Cont, DOCA, and DOCA–RAGE-KO mice (n = 5–6 per group), (**e** and **f**) mesangial matrix expansion and tubulointerstitial fibrosis assessed by periodic acid-Schiff and Masson’s trichrome stains, respectively, in Cont, DOCA, and DOCA–RAGE-KO mice (n = 5 per group), (**g**–**i**) immunohistochemical analysis for glomerular 8-OHdG, GTP-bound Rac1, and MR levels in Cont, DOCA, and DOCA–RAGE KO mice (n = 5 per group), (**j**) glomerular CML staining by immunohistochemistry (n = 4–5 per group), (**k**) podocin expression (n = 4–5 per group), (**l**) cortical mRNA expression levels of MR, SGK1, and TGF-β in Cont, DOCA, and DOCA–RAGE-KO mice (n = 5 per group). Data are presented as the mean ± SEM. ^#^p < 0.05, ^##^p < 0.01 vs. DOCA mice. All sections were 4-μm thin. Bars = 20 μm. RAGE, receptor for advanced glycation end products; Cont, control; WT, wild type; KO, knockout; 8-OHdG, 8-hydroxy-2ʹ-deoxyguanosine; MR, mineralocorticoid receptor; SGK1, serum/glucocorticoid regulated kinase1; TGF-β, transforming growth factor-β.

Renal RAGE expression was significantly upregulated in DOCA mice compared with uninephrectomized salt-administrated mice (Cont mice) (Fig. 2a). We confirmed that RAGE was strongly expressed in the lung of C57BL/6 J (wild type, WT) mice, which was completely deleted in RAGE-knockout (RAGE-KO) mice (Fig. 2b).

### DOCA-Induced Renal Injury was Attenuated in RAGE-KO Mice

We next studied whether activation of MR by DOCA/salt-induced renal damage, which was attenuated in RAGE KO mice. Systolic blood pressure (SBP), blood urea nitrogen (BUN), and creatinine (Cr) levels significantly elevated in DOCA and DOCA–RAGE-KO mice compared with the Cont mice. Although there were no significant differences in terms of SBP (Fig. 2c) and plasma Cr level (Table 1) between the DOCA mice and DOCA–RAGE-KO mice, plasma BUN level tended to be attenuated in DOCA–RAGE-KO mice compared to DOCA mice (p = 0.065) (Table 1). The increases in urinary albumin excretion (UAE), mesangial matrix expansion, and tubulointerstitial fibrosis in DOCA mice were significantly reduced in DOCA–RAGE-KO mice (UAE; 184.3 ± 29.4 vs 47.7 ± 24.2 μg/mg Cr) (Fig. 2d–f). 8-hydroxy-2′-deoxyguanosine (8-OHdG), glomerular CML deposition, Rac1, and MR levels were increased in the kidneys of DOCA mice, all of which were attenuated in DOCA–RAGE-KO mice (Fig. 2g–j). Podocin expression was decreased in the glomeruli of DOCA mice, which was prevented by the genetic deletion of RAGE in DOCA-RAGE-KO mice (Fig. 2k), suggesting that RAGE could be involved in the progression of podocyte injury in MR-activated kidney injury. In addition, enhanced renal gene expression of MR, serine/threonine-protein kinase 1 (SGK1), and transforming growth factor β (TGF-β) were significantly ameliorated in DOCA-RAGE-KO mice (Fig. 2l).

**Table 1:** Clinical characteristics of mice in experiment design.

Parameters	cont	DOCA	DOCA-RAGE-KO	DOCA	
				Ctrl-apt	RAGE-apt
Body weight (g)	23.3 ± 0.6	20.3 ± 0.9*	21.3 ± 0.5*	20.4 ± 0.2	21.6 ± 0.7
KW/BW ratio (mg/g)	9.54 ± 0.5	11.9 ± 0.4*	12.3 ± 0.6*	11.7 ± 0.5	10.1 ± 0.3^#^
Serum BUN (mg/dL)	30.7 ± 0.7	57.9 ± 3.1*	48.6 ± 3.8^*‡^	60.0 ± 4.5	63.8 ± 3.6
Serum Cr (mg/dL)	0.10 ± 0.01	0.18 ± 0.02*	0.18 ± 0.01*	0.17 ± 0.02	0.20 ± 0.01

Data are mean ± SEM. *p < 0.05 vs. Ctrl, ^#^p < 0.05 vs DOCA-Ctrl-apt mice, ^‡^p = 0.065 vs DOCA.

RAGE: receptor for advanced glycation end products, KO: knockout, Ctrl: control, apt: aptamer, KW: kidney weight, BW: body weight, BUN: blood urea nitrogen, Cr: creatinine.

### Characterization of RAGE-aptamers

**Figure 3:**
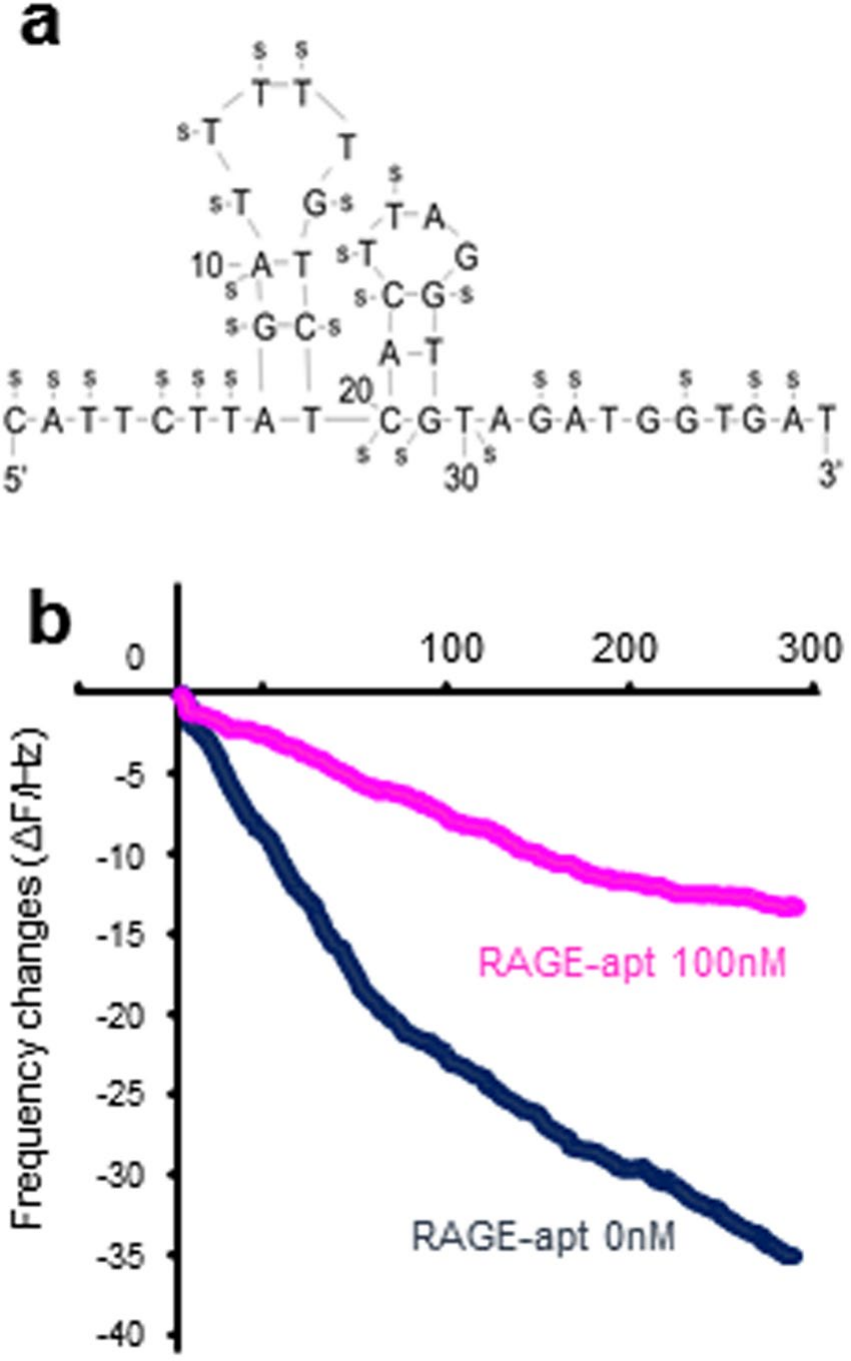
(**a**) Structure of #2 RAGE-apt, (**b**) RAGE-apt (100 nM) blocks the binding of CML to vRAGE assessed by QCM method. RAGE, receptor for advanced glycation end products; RAGE-apt, RAGE-aptamer; Ctrl-apt, control-aptamer; ELISA, enzyme-linked immunosorbent assay; CML-BSA, carboxymethyllysine-bovine serum albumin; QCM, quartz crystal microbalance.

We selected 7 RAGE-apts by a Systematic Evolution of Ligands using the EXponential enrichment (SELEX) method as previously described^18^. We selected #2 RAGE-apt and used it in the following experiments because of its strongest antagonistic ability for RAGE. #2 RAGE-apt has double bulge loops (Fig. 3a), which may be important for binding to the v-domain of the human RAGE (v-RAGE). A sensitive 27-MHz quartz crystal microbalance (QCM) also revealed that #2 RAGE-apt bound to v-RAGE with a dissociation constant (Kd) of 0.1 nM; RAGE-apt markedly blocked the binding of CML-BSA to v-RAGE (Fig. 3b). We also found that CML-BSA bound to v-RAGE with a Kd of 28.3 nM.

### Effects of RAGE-aptamer on Renal Injury in DOCA mice

**Figure 4:**
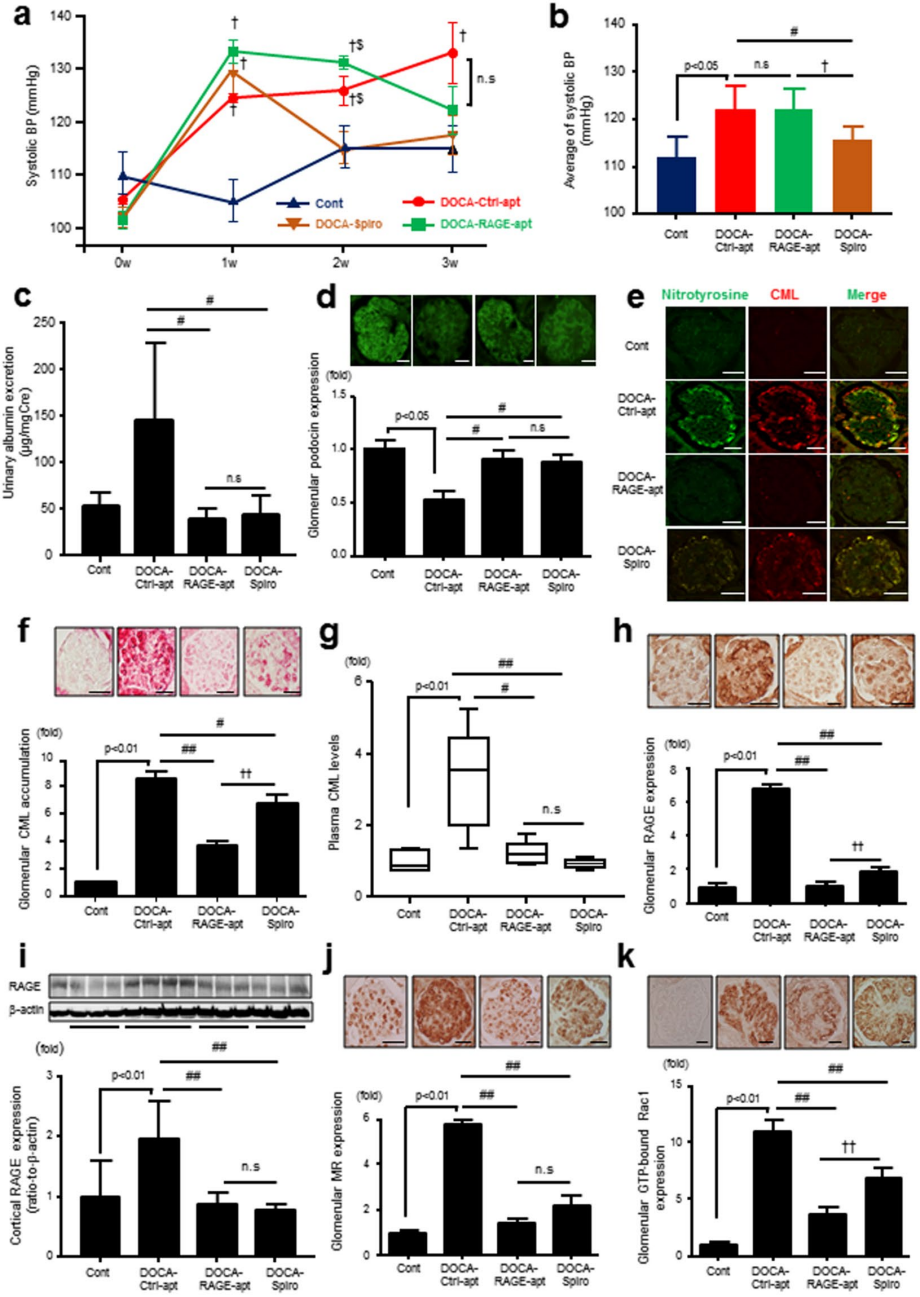
RAGE-apt ameliorates the progression of MR-associated podocyte injury through the inhibition of oxidative stress production and Rac1 activation. (**a** and **b**) SBP levels in Cont, DOCA-Ctrl-apt, DOCA–RAGE-apt, and DOCA-Spiro mice (n = 4–12 per group), ^†^p < 0.05 vs Cont, ^$^p < 0.05 vs DOCA-Spiro, (**c**) UAE levels (n = 4–12 per group), (**d**) podocin expression (n = 4–5 per group), (**e**) double staining for nitrotyrosine and CML by immunofluorescence (n = 4–5 per group), (**f**) glomerular CML staining by immunohistochemistry (n = 4–5 per group), (**g**) plasma CML levels (n = 4–12 per group), (**h**) glomerular RAGE expression (n = 4–5 per group), (**i**) cortical RAGE protein expression by Western blot (n = 4 per group), (**j** and **k**) glomerular GTP-bound Rac1 and MR expression (n = 4–5 per group). Data are presented as mean ± SEM. ^#^p < 0.05, ^##^p < 0.01 vs DOCA-Ctrl-apt mice, ^†^p < 0.05, ^††^p < 0.01 vs DOCA-Spiro mice. All kidney sections are 4-μm thin. Bars = 20 μm. RAGE, receptor for advanced glycation end products; MR, mineralocorticoid receptor; SBP, systolic blood pressure; Cont, control; Ctrl-apt, control aptamer; RAGE-apt, RAGE aptamer; UAE, urinary albumin excretion; Spiro, spironolactone; CML, carboxymethyllysine.

Although the continuous administration of RAGE-apt did not affect SBP in DOCA mice, it reduced the increase in UAE (Fig. 4a–c). As with DOCA-RAGE-KO mice, podocin expression decreased in DOCA-Ctrl-apt–treated mice, which was restored in RAGE-apt–treated DOCA mice (Fig. 4d). Nitrotyrosine (NT) was co-localized with CML in the podocytes of DOCA-Ctrl-apt mice, and their levels increased in DOCA-Ctrl-apt–treated mice, both of which were suppressed by RAGE-apt treatment (Fig. 4e). The increases in glomerular CML, RAGE, MR, and Rac1 expressions as well as plasma CML and cortical RAGE levels in DOCA-Ctrl-apt–treated mice were also significantly reduced by RAGE-apt or spironolactone (Spiro) (Fig. 4f–k).

### Aldosterone Induces CML, ROS, and RAGE Production in Murine Podocyte Cells (MPC)

**Figure 5:**
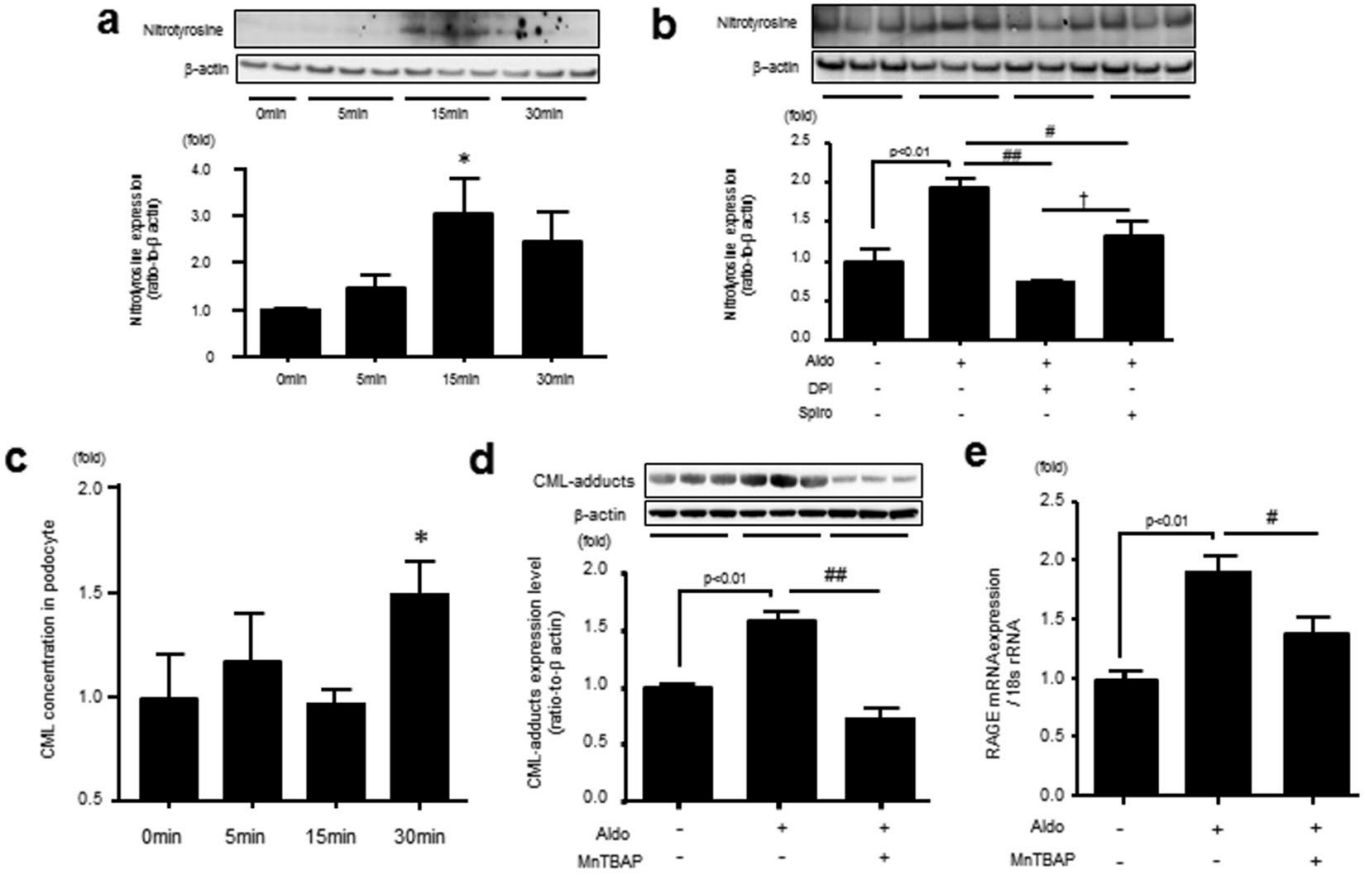
Aldo–MR system enhances nitrotyrosine and CML production through oxidative stress generation in MPC. (**a**) MPC were incubated with Aldo (1 µM) for 5, 15, and 30 min, and nitrotyrosine expression, a marker of peroxynitrite, was measured by Western blot (n = 3). (**b**) MPC were incubated with Aldo with or without DPI or Spiro for 15 min, and nitrotyrosine was measured by Western blot in MPC (n = 6). (**c** and **d**) MPC were incubated with Aldo for 30 min, then intracellular CML (ELISA) and CML (Western blot) expression were determined with or without MnTBAP, an anti-oxidant, in MPC (n = 6). (**e**) Aldo was administrated for 4 h, and RAGE mRNA expression were measured with or without MnTBAP by RT-PCR in MPC (n = 6). Data are presented as mean ± SEM. *p < 0.05 vs 0 min, ^#^p < 0.05, ^##^p < 0.01 vs Aldo. Aldo, aldosterone; MR, mineralocorticoid receptor; CML, carboxymethyllysine; MPC, mouse podocyte cells; DPI, diphenyleneiodonium; MnTBAP, manganese (III) tetrakis (4-benzoic acid) porphyrin chloride; RAGE, receptor for advanced glycation end products; RT-PCR, real-time polymerase chain reaction.

Aldo increased NT levels, which was suppressed by pretreatment with diphenylene iodonium (DPI), an inhibitor of NADPH oxidase, or Spiro (Fig. 5a and b). Furthermore, Aldo increased intracellular CML levels and RAGE gene expression in MPC, both of which were reduced by the anti-oxidant manganese (III) tetrakis (4-benzoic acid) porphyrin chloride (MnTBAP) (Fig. 5c–e)^19,20^.

### RAGE-aptamer Improves CML-BSA-elicited MR and GTP-bound Rac1 Expressions in MPC

**Figure 6:**
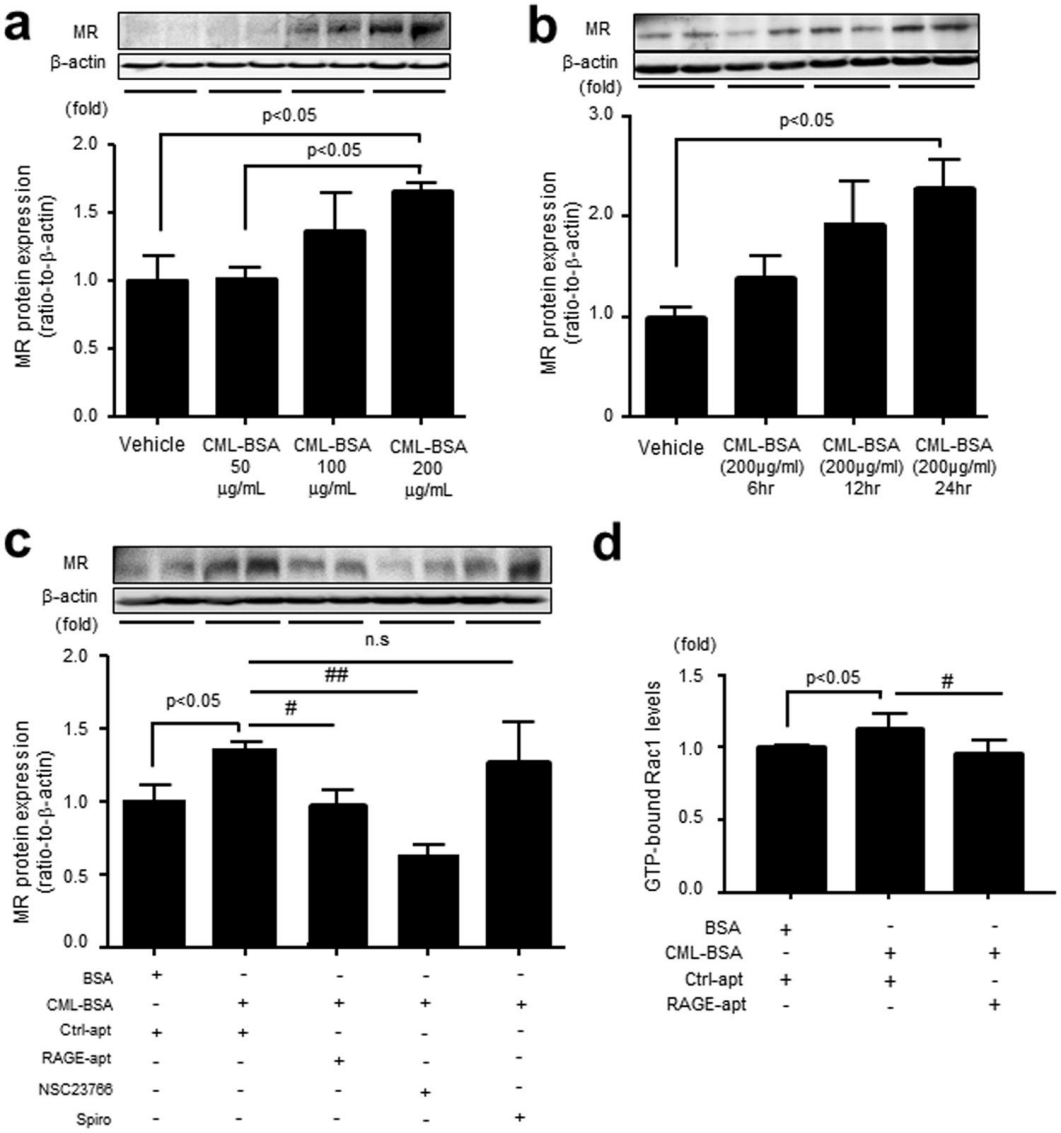
CML-BSA increases MR expression through RAGE–Rac1 axis in MPC. (**a**) MPC were incubated with CML-BSA (50, 100, and 200 μg/ml) for 24 h, and MR expression was determined by Western blot (n = 4, respectively). (**b**) CML-BSA (200 μg/ml) was administrated for 6, 12, and 24 h, and MR expression was determined (n = 4, respectively). (**c**) MPC were co-incubated with CML-BSA or BSA (200 μg/ml) with or without Ctrl-apt (100 nM), RAGE-apt (100 nM), NSC23766 (100 μM), a Rac1 inhibitor, or Spiro (10 μM) for 24 h, and MR expression was evaluated by Western blot (n = 5). (**d**) CML-BSA or BSA (200 μg/ml) was co-incubated with Ctrl- or RAGE-apt (100 nM) for 5 min in MPC, and Rac1 activation was determined (n = 4). Data are presented as the mean ± SEM. ^#^p < 0.05, ^##^p < 0.01 vs CML-BSA. CML-BSA, carboxymethyllysine-bovine serum albumin; MR, mineralocorticoid receptor; RAGE, receptor for advanced glycation end products; MPC, mouse podocyte cells; BSA, bovine serum albumin; Ctrl-apt, control aptamer; RAGE-apt, RAGE aptamer; Rac1, GTP-bound Rac1; Spiro, spironolactone.

**Figure 7:**
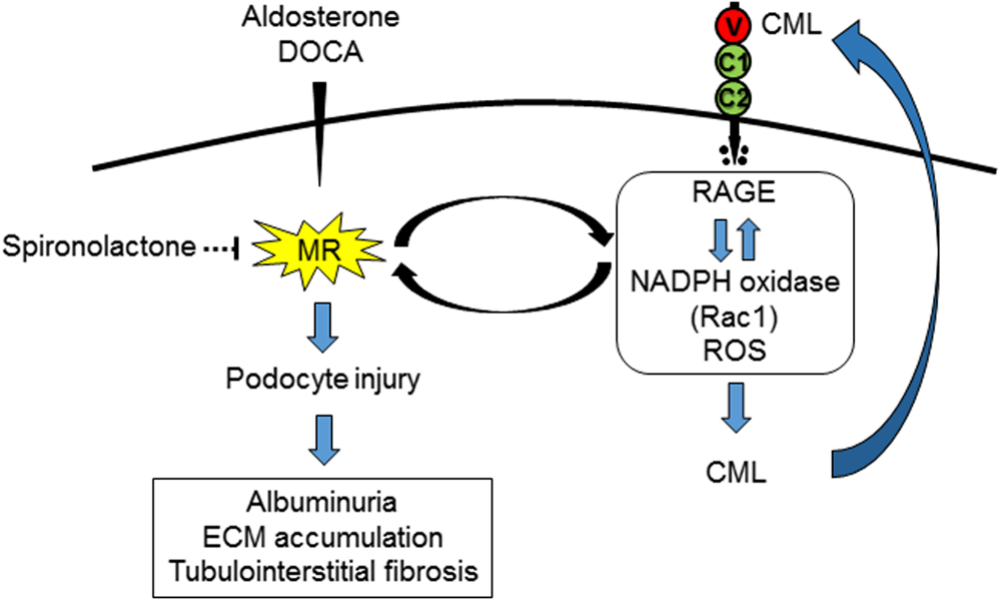
Hypothetic pathways of Aldo–MR system and AGE–RAGE axis-mediated podocyte injury in hypertensive nephropathy. MR, mineralocorticoid receptor; ECM, extracellular matrix; CML, carboxymethyllysine; RAGE, receptor for advanced glycation end products; ROS, reactive oxygen species.

CML-BSA increased MR levels in a dose- and time-dependent manner in MPC. RAGE-apt, NSC23766, an inhibitor of Rac1, but not Spiro, significantly blocked the increase in MR expression in MPC (Fig. 6a–c). In addition, CML-BSA enhanced the activation of Rac1 in MPC (Fig. 6d). Taken together, these data indicate that inhibition of AGE-RAGE axis can block podocyte injury induced by MR activation (Fig. 7).

## Discussion

We showed for the first time that although the administration of RAGE-apt for 3 weeks did not affect blood pressure levels, it reduced UAE, restored podocin expression, and inhibited the increases in glomerular CML, RAGE, MR, and Rac1 levels in DOCA mice. CML and NT were co-localized and increased in the podocytes of the glomeruli of DOCA mice, which was also prevented by RAGE-apt. Since Spiro mimicked the effects of RAGE-apt, the present study suggests that DOCA could stimulate the AGE–RAGE axis in podocytes of the glomeruli through the interaction with MR via the Rac1 pathway, thereby leading to podocyte damage and increased UAE in DOCA mice.

Podocyte dysfunction and loss are one of the characteristic features of several renal diseases^21–23^. AGE and RAGE are reported to be strongly expressed in glomerular sclerosis lesions in patients with hypertension^24^. Further, RAGE is dominantly overexpressed in podocyte^24^, thus suggesting the clinical relevance of AGE–RAGE axis in podocyte injury in hypertensive renal disease. Recently, we have found that RAGE-neutralizing antibody inhibits the AGE-induced upregulation of RAGE mRNA levels, oxidative stress generation, and apoptosis in podocytes^25^. Therefore, the interaction of AGE with RAGE per se could modulate RAGE expression via the production of intracellular reactive oxygen species (ROS) as a second messenger. Since oxidative stress is generated by MR-mediated Rac1 overexpression and ROS stimulates intracellular AGE production in podocytes^26,27^, DOCA-elicited MR activation may enhance ROS generation and subsequently promote intracellular AGE production, which could elicit podocyte injury through interaction with RAGE. Nitric oxide is shown to rapidly react with superoxide anion radical derived from NADPH oxidase, forming ONOO- in kidney cells^28^. Indeed, AGE induced the production of inducible nitric oxide synthase (iNOS) and accelerated ONOO- production through the interaction with RAGE in diabetic condition^29,30^. That could be the reason why RAGE-apt inhibited the expression levels of nitrotyrosine in the podocytes of DOCA mice. Therefore, we hypothesize that nitric oxide may play a crucial role for ONOO- production, leading to podocyte injury through the crosstalk with RAGE in DOCA-induced kidney injury.

In the present study, genetic RAGE deletion also significantly suppressed DOCA-induced increase in MR production, Rac1 activation, CML deposition, and the downstream signaling of SGK1 and TGF-β. These findings are in accordance with the observations of RAGE-apt experiments showing that RAGE-apt could prevent DOCA-elicited renal injury via the Rac1–ROS–AGE pathway. Further, we observed that Aldo significantly increased NT and CML expressions in association with increased RAGE gene expression in MPC. Since CML accumulation in atherosclerotic plaques was diminished in RAGE-deleted ApoE-KO diabetic mice^31^, it is conceivable that engagement of Aldo-elicited CML to RAGE stimulates ROS production, which in turn promotes the further production of CML, thus forming a positive feedback loop between RAGE downstream signaling and CML generation. Moreover, CML increased MR expression and Rac1 activation, which were blocked by the RAGE-apt in MPC. These findings suggest that MR expression and AGE–RAGE system are closely correlated with each other, leading to podocyte injury (Fig. 7). DOCA-induced mesangial matrix expansion and tubulointerstitial fibrosis were restored in RAGE-KO mice (Fig. 2e–f). In addition, glomerular matrix accumulation was ameliorated by the treatment with RAGE-apt (Supplementary Figure S3). Since DOCA-elicited SGK1 and TGF-β expression were ameliorated in RAGE-KO mice, RAGE-apt might restore the MR-induced glomerular matrix accumulation through the suppression of SGK1 and TGF-β expression, thereby protecting damages in several renal cells including podocytes, mesangial cells, and proximal tubular cells.

In the present study, we observed that RAGE-apt did not affect plasma BUN and Cr levels when compared to DOCA-Ctrl-apt mice. On the basis of the result showing that genetic deletion of RAGE tended to improve the plasma BUN level (p = 0.065, Table 1), the pharmacokinetics of RAGE-apt such as bioavailability and dissociation constant might be associated with the failure to attenuate plasma BUN and Cr levels in DOCA-mice. Secondly, we believe the polyuria associated with the DOCA model may mask some protective effects as measured by serum markers of kidney function. It is generally accepted that urine volume was increased in DOCA/salt-treated mice, which was reduced by normalization of blood pressure using hydralazine, a direct acting vasodilator without affecting any urine electrolytes^32^, suggesting that increased blood pressure could be strongly associated with urine volume and subsequent dehydration-induced renal function. In the present study, we observed that the administration with Spiro could reduce the elevated systolic blood pressure in DOCA mice (Fig. 4b) and significantly decrease their urine volume (DOCA-Ctrl-apt vs DOCA-Spiro; 2.47 ± 0.27 vs 1.37 ± 0.12 (mL), p < 0.01). On the other hand, the pharmacological deletion of RAGE using RAGE-apt did not affect systolic blood pressure (Fig. 4b) and the amount of urine volume (DOCA-Ctrl-apt vs DOCA-RAGE-apt; 2.47 ± 0.27 vs 2.71 ± 0.21 (mL), p = 0.51). We hypothesize that polyuria could lead to dehydration, minimizing the apparent protective effect of RAGE-apt as measured by serum markers of kidney function in spite of their structural improvement. In addition, histological changes are sometimes more sensitive than markers of kidney function for detecting kidney injury in early phase of chronic kidney disease. Therefore, we have concluded that RAGE-apt could be a promising therapeutic agent against MR-activated kidney injury.

There is a growing body of evidence that MR antagonism using Spiro or eplerenone can reduce urinary albumin excretion and retard the progression of chronic kidney disease in several clinical studies^33,34^. We hypothesize that Spiro is an excellent combination therapy to use in combination with RAGE-apt in MR-activated kidney injury. Our *in vitro* data suggested that aldosterone-induced nitrotyrosine production was reduced by the treatment with Spiro (Fig. 5b), but it does not completely block the CML-induced MR activation (Fig. 6c). In addition, we found that the CML deposition in the kidney tissue was partially, but significantly, improved by the treatment with Spiro *in vivo*, though not to the same extent as RAGE-apt (Fig. 4f). These findings suggest that RAGE-apt and Spiro target independent pathways in the progression of podocyte injury, thereby the combination therapy of Spiro plus RAGE-apt can be promising treatment against MR-activated kidney injury.

Several therapeutic agents targeting RAGE have been reported^12,35,36^. Administration of soluble RAGE suppressed the progression of diabetic atherosclerosis in ApoE-KO mice^35^. Long-term treatment with neutralizing RAGE antibody ameliorated renal injury in obese type 2 diabetic mice model^36^. However, due to several limitations, these agents have not been utilized in clinical setting. On the contrary, RAGE-apts have several advantages over soluble RAGE protein or RAGE antibody. First, the synthesis of aptamers does not rely on animal systems; therefore, they can be easily selected from oligonucleotide library *in vitro*. Second, aptamers are quite thermally stable, whereas proteins are sensitive to temperature. Third, aptamers do not have immunogenicity over soluble proteins or antibodies. Finally, a small size allows for more efficient entry of RAGE-apt into various organs^37,38^. Recently, we found that AGE-DNA-apt significantly inhibited the progression of experimental diabetic nephropathy^17^. However, since AGEs are composed of complex and heterogeneous compounds^11^, targeting one type of AGEs may not sufficiently block the AGE–RAGE system. Therefore, RAGE-apt may be a more favorable tool for blockade of the AGE–RAGE axis. Although some aptamers targeting receptor may have an agonistic function^39^, our RAGE-apt did not show any agonistic activity of RAGE because RAGE-apt alone did not affect the gene expression of RAGE, connective tissue growth factor, and monocyte chemokine protein-1 (Supplementary Figure S4). Therefore, our RAGE-apt could be safe and effective for the treatment of several renal injury, including HN^12^.

## Concise Methods

### Animal Preparation

Eight-week-old male WT mice were purchased from CLEA Japan (Tokyo, Japan). The RAGE-KO mouse strains on C57BL/6J background were created by Professor Arnold (Deutsches Krebsforschungszentrum Stiftung des öffentlichen Rechts, Heidelberg, Germany) and Professor Nawroth (University Clinical Centre of Heidelberg) and were kindly given from Dr. Bierhaus (Deutsches Krebsforschungszentrum Stiftung des öffentlichen Rechts, Heidelberg, Germany)^40,41^.

Hypertension was induced in C57BL/6J and RAGE-KO mice by uninephrectomy with the administration of DOCA (50 mg, a 21-day continuous-release, Innovation Research, USA) and 4% salt diet, with the following categories: 4% salt diet, Cont; 4% salt diet with DOCA, DOCA; RAGE-KO mice treated with DOCA/salt, DOCA–RAGE-KO. RAGE-apt (2.0 × 10^−4^ μg/day) or Ctrl-apt (2.0 × 10^−4^ μg/day) were administrated in DOCA mice subcutaneously by an osmotic pump (model 2006; ALZET, Cupertino, USA). Spiro (30 mg/kg per day) was administrated by a feeding needle. All experimental procedures were conducted in accordance with the National Institutes of Health Guide for the Care and Use of Laboratory Animals and were approved by the ethics committee of Kurume University School of Medicine.

### Measurement of Clinical Variables

Albuminuria was determined with a commercially available ELISA kit (Exocell, Philadelphia, USA). Blood was collected, and the plasma was stored at −40 °C. BUN and Cr levels in the plasma were measured by an enzymatic method using an auto-analyzer (LABOSPECT 008; Hitachi, Tokyo, Japan).

### Preparation of CML-BSA

CML-BSA was prepared by a reaction with glyoxylic acid with BSA in the presence of NaBH3CN, as previously described^42^.

### Screening and Blocking Capacity of RAGE–DNA Aptamer

RAGE-apt was obtained by an *in vitro* selection process, that is, SELEX methods, from a pool of ~10^15^ different nucleic acid sequences as previously described^18^. Seven types of sequences directed against RAGE were obtained in this study and sequences of selected RAGE-apts and Ctrl-apt are displayed in Supplementary Figure S5a. These aptamers were modified with phosphorothioate for protection from degradation by nuclease^43^. We examined the blocking capacity of RAGE-apts between CML-BSA and v-RAGE by ELISA. v-RAGE (residues 23–121) was prepared as previously described^44,45^. In brief, CML-BSA was immobilized on the bottom of 96 well-ELISA plate, and v-RAGE was added with RAGE- or Ctrl-apts. CML-BSA-bound v-RAGE was captured by horseradish peroxidase-conjugated antibody against v-RAGE and then detected via an enzyme-catalyzed color reaction. Clone #2 showed the highest binding affinity to CML-BSA (Supplementary Figure S5b). Next, to confirm the inhibitory capacity of #2 RAGE-apt against the interaction of CML-BSA with RAGE, we performed sensitive 27-MHz QCM (Ainix Q, Intium, Tokyo, Japan)^46^. In brief, v-RAGE was immobilized on an avidin-bound QCM surface. After adding CML-BSA to a reaction vessel with or without RAGE-apt (100 nM), the time course of the frequency decrease on the QCM was monitored.

### Immunohistochemical Analysis

Specimens of kidney cortex were fixed with 4% paraformaldehyde, embedded in paraffin, sectioned at 4-µm intervals, and mounted on glass slides. The sections were incubated with antibodies (Abs) raised against MR (1:100), RAGE (1:100) (kindly provided by Professor Yamamoto Y, Kanazawa University Graduate School of Medical Science, Kanazawa, Japan), CML (1:1000), 8-OHdG (1:1000), and Rac1 (1:100) after pretreatment with blocking agent (Supplementary Figure S1). The sections were incubated with Envision HRP-labeled polymer anti-rabbit and mouse (ready-to-use) (Dako, cat# K536111–2). Immunoreactivity in 20 different fields (×600) in each sample was evaluated by image analysis software (version 6.57; Optimas, Media Cybernetics, Silver Spring, MD).

### Immunofluorescence Study by Confocal Microscopy

To determine the localization of RAGE, MR, and Rac1 in the glomeruli of DOCA mice, we performed an immunofluorescence staining with Abs raised against RAGE (1:100), MR (1:100), Rac1 (1:100), and WT-1 (1:1000) as a primary Ab (Supplementary Figure S1). Goat anti-rabbit IgG Alexa 488 and anti-mouse IgG Alexa 594 (1:1000; Molecular Probes, Eugene, OR, USA) were used as secondary Abs. The sections were analyzed under a confocal laser microscope, FV 1000 (OLYMPUS, Tokyo, Japan). Double staining of CML and NT was also performed using anti-CML and anti-NT Abs as a primary antibody. Podocin was evaluated by immunofluorescence study with anti-podocin Ab (1:1000, kindly provided by Dr. Asanuma K) as a primary Ab and goat anti-mouse IgG Alexa 488 (1:1000) as a secondary Ab to investigate podocyte dysfunction.

### Morphological Analysis

Four-µm paraffin sections were stained with periodic acid-Schiff and Masson’s trichrome for light microscopic analysis^47^.

### Measurement of CML in Plasma and Cell Lysate

CML levels in plasma and cell lysate were measured using a competitive ELISA according to the manufacturer’s instructions (Cell Biolabs, Inc. cat #STA-816).

### Cells

Conditionally immortalized MPC were kindly provided by Dr. Asanuma K, and cultured as previously described^25,48^. MPC was treated with Aldo (1 µM), CML-BSA (50–200 µg/ml) or non-glycated BSA (200 µg/ml) with RAGE-apt (100 nM), Ctrl-apt (100 nM), NSC23766 (100 µM), Spiro (10 µM), and MnTBAP (10 µM) in a medium containing 1% fetal bovine serum.

### Western Blot

Kidney, lung, and MPC were lysed, and lysates were prepared as previously described^49^. Membranes were incubated with the following primary Abs: RAGE, NT, CML, MR, SGK1, and GAPDH (1:1000, respectively) and β-actin (1:2000) during overnight (Supplementary Figure S1). Horseradish peroxidase-conjugated anti-mouse and anti-rabbit secondary antibodies (1:2000) were applied. Protein expressions were visualized by Signal Enhancer HIKARI (Nacalai Tesque, Inc., Kyoto, Japan).

### Real-Time RT-PCR

Total RNAs were extracted from each kidney cortex and MPC with TRIzol reagent (Invitrogen, USA), and isolated RNAs were used to synthesize cDNA with Rever Tra Ace qPCR RT Master Mix with gDNA Remover (Toyobo, Tokyo, Japan). Quantitative real-time RT-PCR was performed using Assay-on-Demand and Taqman 5 fluorogenic nuclease chemistry (Applied Biosystems, USA). Information of primers and probes are shown in Supplementary Figure S2. The relative mRNA expressions of each gene were calculated with ∆∆Ct method.

### Rac1 Activity by G-LISA

For evaluation of GTP-bound Rac1 activity, the Rac1 G-LISA Activation Assay Biochem Kit (Cytoskeleton, Denver, CO) was performed according to the manufacture’s instruction. In brief, MPC were placed on ice and washed with ice-cold PBS after the incubation with CML-BSA (200 μg/mL) or BSA (200 μg/mL) for 5 min in the presence or absence of RAGE-apt. MPC were harvested with ice-cold lysis buffer, then lysates were added to the wells of the Rac1-GTPase binding plate. After addition of primary and horseradish peroxidase-conjugated secondary Abs, signals were measured using a microplate luminometer (2030 ARVO x3, Perkin Elmer).

### Statistical Analyses

All data are presented as mean ± SEM. One-way ANOVA and following unpaired t-test were used to assess the differences among groups when appropriate. All statistical analyses were performed using GraphPad Prism 7 Software (GraphPad Software, San Diego, CA). p < 0.05 was considered statistically significant.

## Electronic supplementary material

Supplementary data
