# Supplementary material for: RAGE-aptamer attenuates deoxycorticosterone acetate/salt-induced renal injury in mice
Source: Sci Rep. 2018 Feb 8;8:2686. doi: 10.1038/s41598-018-21176-5 (PMC5805738; doi:10.1038/s41598-018-21176-5)
Supplement: Supplementary file 1 — Supplementary data [file 41598_2018_21176_MOESM1_ESM.docx]

**RAGE-aptamer attenuates deoxycorticosterone acetate/salt-induced renal injury in mice**

Kensei Taguchi^1,6^, Sho-ichi Yamagishi^2^, Miyuki Yokoro^1,3^, Sakuya Ito^1^, Goh Kodama^1^, Yusuke Kaida^1^, Yosuke Nakayama^1^, Ryotaro Ando^1^, Nana Yamada-Obara^1^, Katsuhiko Asanuma^4^, Takanori Matsui^2^, Yuichiro Higashimoto^5^, Craig R Brooks^6^, Seiji Ueda^1,7^, Seiya Okuda^1^, and Kei Fukami^1^

^1^ Division of Nephrology, Department of Medicine, Kurume University School of Medicine, Kurume, Japan

^2^ Department of Pathophysiology and Therapeutics of Diabetic Vascular Complications, Kurume University School of Medicine, Kurume, Japan

^3^ Department of Food Sciences and Nutrition, School of Human Environmental Sciences, Mukogawa Women’s University, Nishinomiya, Japan

^4^ Department of Nephrology, Chiba University Graduate School of Medicine, Chiba, Japan

^5^ Department of Medical Biochemistry, Kurume University School of Medicine, Kurume, Japan

^6^ Division of Nephrology, Department of Medicine, Vanderbilt University Medical Center, Nashville, Tennessee, USA

^7^ Division of Nephrology, Department of Internal Medicine, Juntendo University, Tokyo, Japan

**Address for correspondence**:

Kei Fukami, MD, PhD

Division of Nephrology, Department of Medicine

Kurume University School of Medicine

67 Asahi-machi, Kurume, Japan

Postal code; 830-0011

Email: fukami@med.kurume-u.ac.jp

Tel: +81-942-31-7002

Fax: +81-942-31-7763

**Supplementary Figures and Tables**

**Supplementary Figure S1**

**
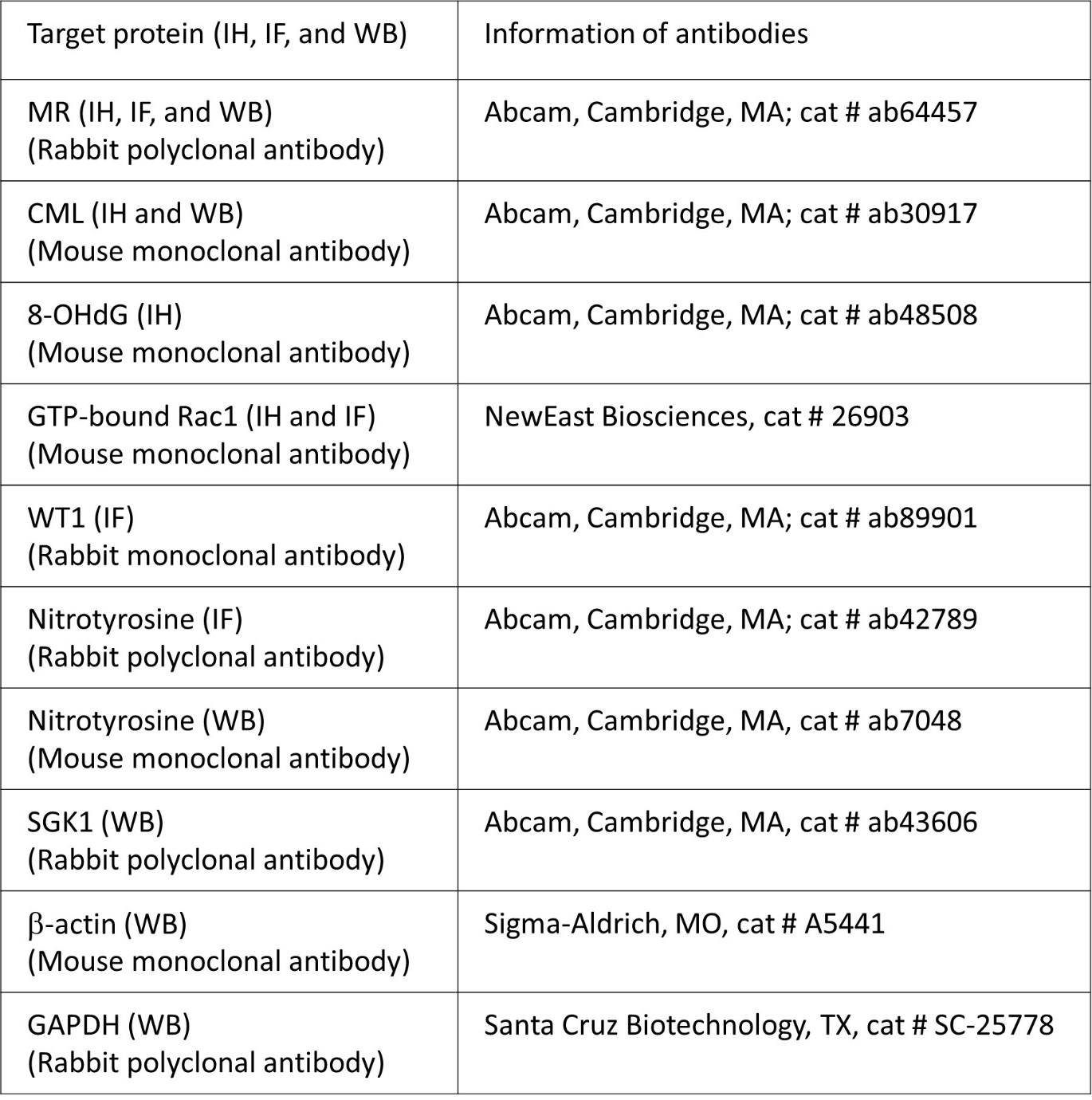
**

**Supplemental Figure 1**

The information of antibodies for IH, IF, and WB. IH, immunohistochemical analysis; IF, immunofluorescence analysis; WB, western blotting; MR, mineralocorticoid receptor; CML, carboxymethyllysine; 8-OHdG, 8-hydroxy-2ʹ-deoxyguanosine; WT1, Wilms’ tumor 1, SGK1, serum/glucocorticoid regulated kinase1; GAPDH, glyceraldehyde-3-phosphate dehydrogenase.

**Supplementary Figure S2**

**
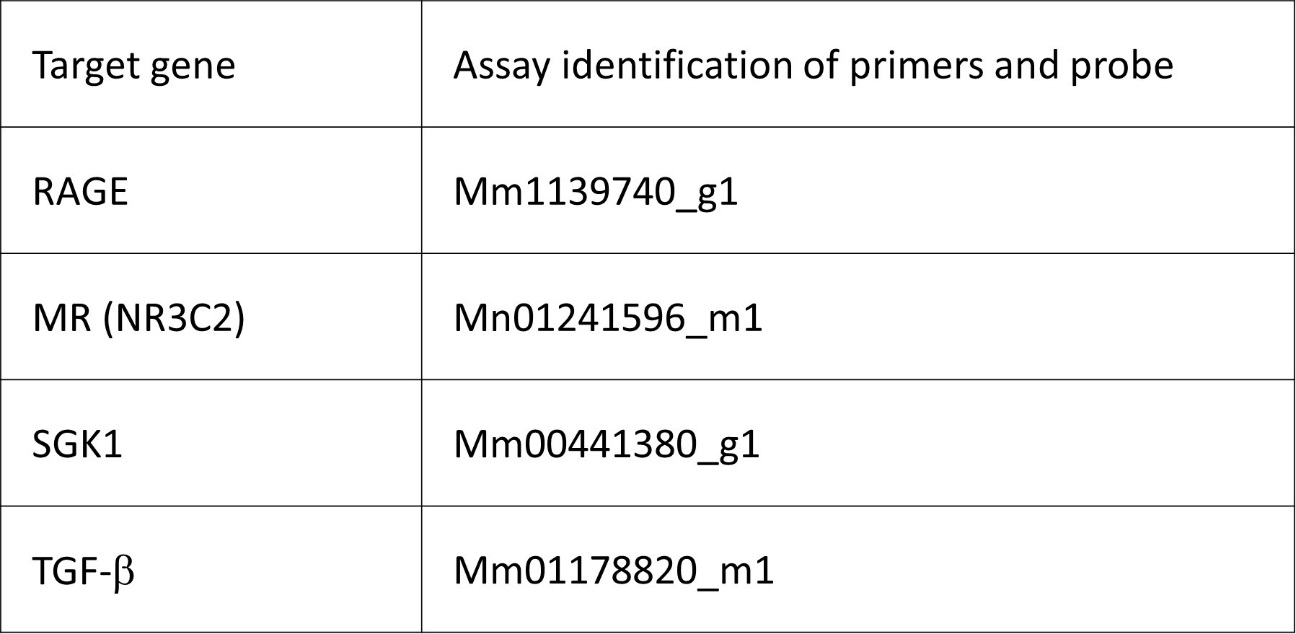
**

**Supplemental Figure 2**

Assay identification of primers and probe for real-time PCR. RAGE, receptor for advanced glycation end products; MR, mineralocorticoid receptor; SGK1, serum/glucocorticoid regulated kinase1; TGF-β, transforming growth factor β; PCR, polymerase chain reaction.

**Supplementary Figure S3**


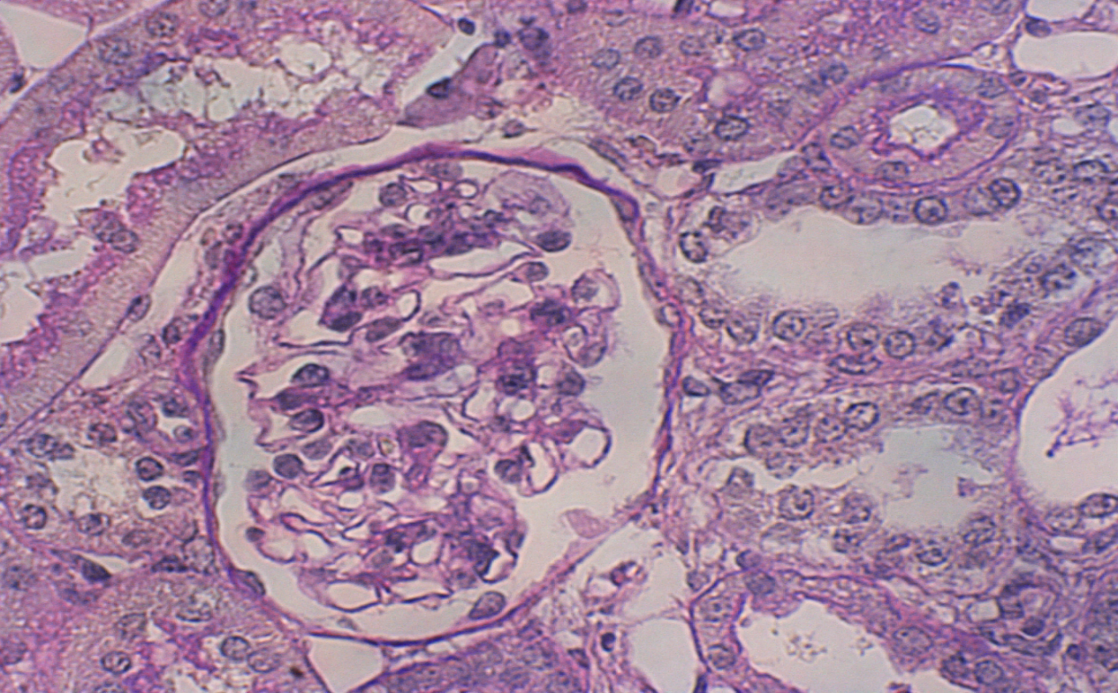

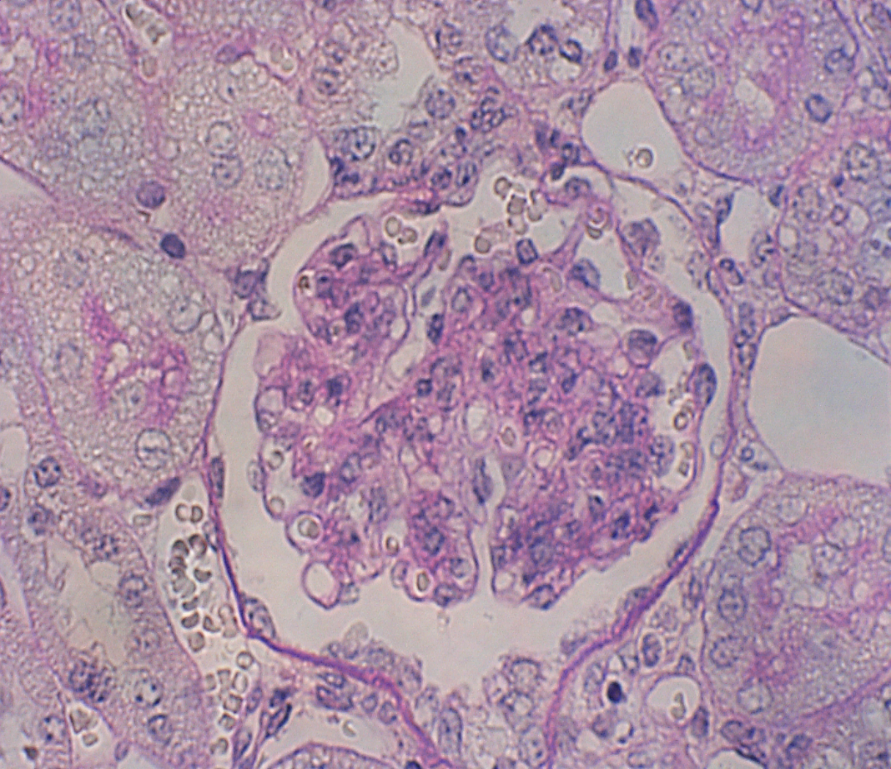


DOCA+Ctrl-apt

DOCA+RAGE-apt


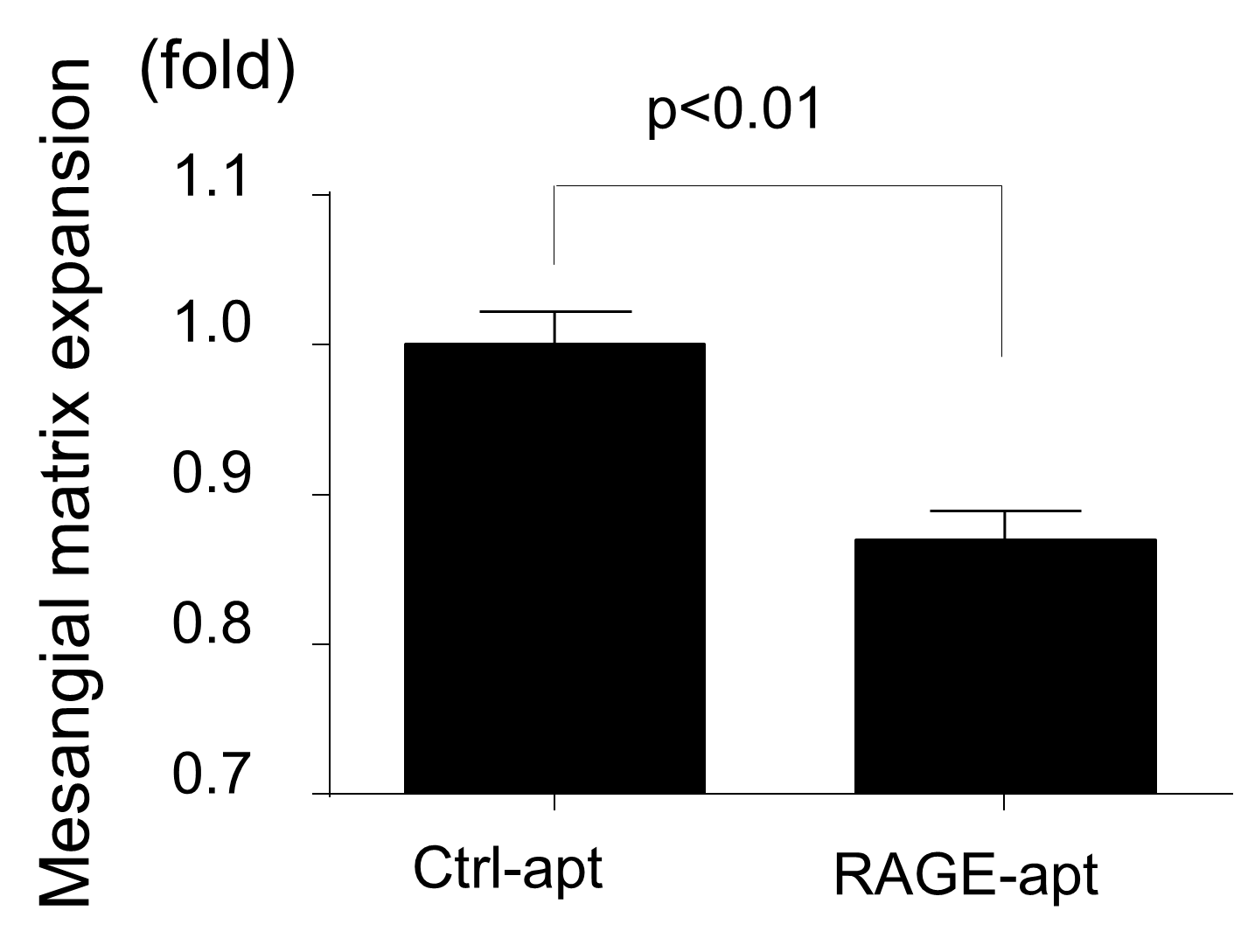


**Supplementary Figure S3.**

**RAGE-apt reduces the DOCA-induced mesangial matrix expansion.**

Mesangial matrix expansion was increased in the glomeruli of DOCA-induced hypertensive mice, which was significantly attenuated by the treatment with RAGE-apt. kidney sections are 4-μm thin. Bars = 20 μm. RAGE, receptor for advanced glycation end products; Ctrl-apt, control aptamer; RAGE-apt, RAGE aptamer.

**Supplementary Figure S4**

**
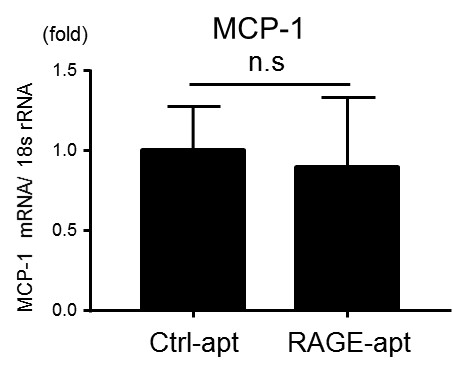
**

**
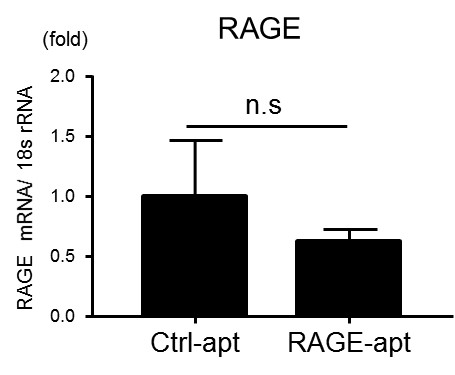
**

**
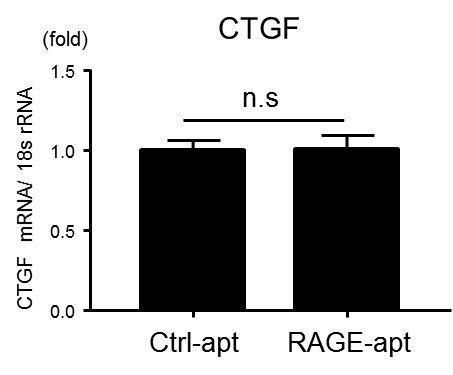
**

**Supplementary Figure S4.**

**RAGE-apt per se did not induce profibrotic and proinflammatory gene expression on Human Renal Proximal Tubule Epithelial Cells.**

Human Renal Proximal Tubule Epithelial Cells (RPTECs) were cultured with Ctrl-apt or RAGE-apt for 24 h. The incubation with RAGE-apt did not upregulate the messenger RNA expression of RAGE, MCP-1, and CTGF, indicating that RAGE-apt has no agonist efficacy. RAGE, receptor for advanced glycation end products; MCP-1, Monocyte Chemotactic Protein-1; CTGF, Connective Tissue Growth Factor; Ctrl-apt, control aptamer; RAGE-apt, RAGE aptamer.

**Supplementary Figure S5**

**a**

**
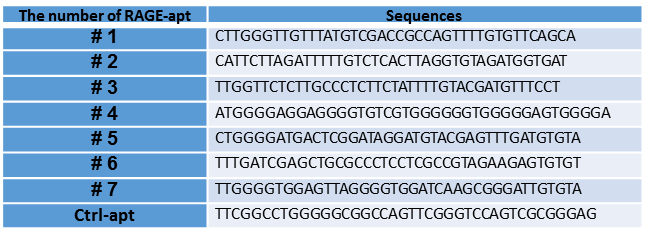
**

**b**

**
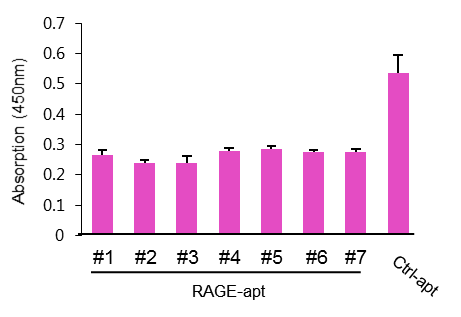
**

**Supplementary Figure S5.**

(a) Sequences of 7 RAGE-apts and Ctrl-apts and (b) the blockade capacity of RAGE-apts by ELISA. All RAGE-apt block the binding of CML-BSA to v-RAGE, RAGE, receptor for advanced glycation end products; RAGE-apt, RAGE-aptamer; Ctrl-apt, control-aptamer; ELISA, enzyme-linked immunosorbent assay. Supplementary figure S5a shows the sequences of RAGE-apts and control-aptamer (Ctrl-apt). All RAGE-apts significantly blocked the binding of carboxymethyllysine (CML)-bovine serum albumin (BSA) to RAGE by an enzyme-linked immunosorbent assay (ELISA) (Supplementary figure S5b).

**Supplementary Figure S6**

**a**

**
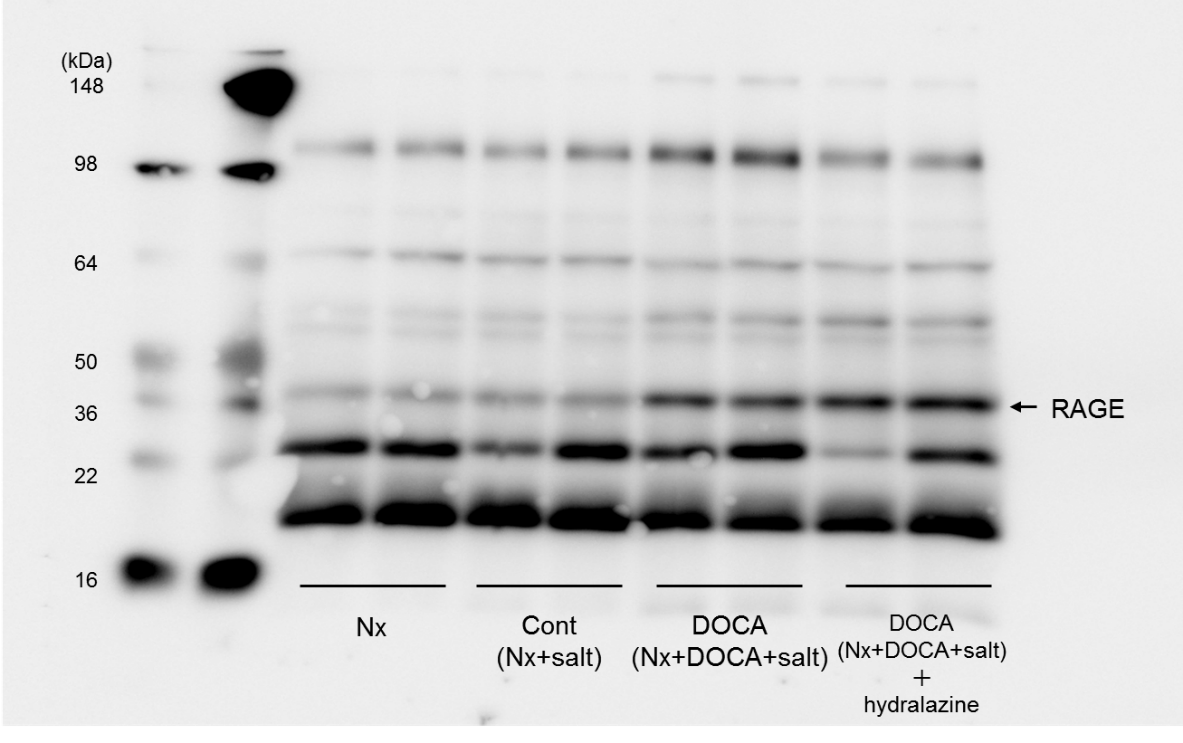
**

**
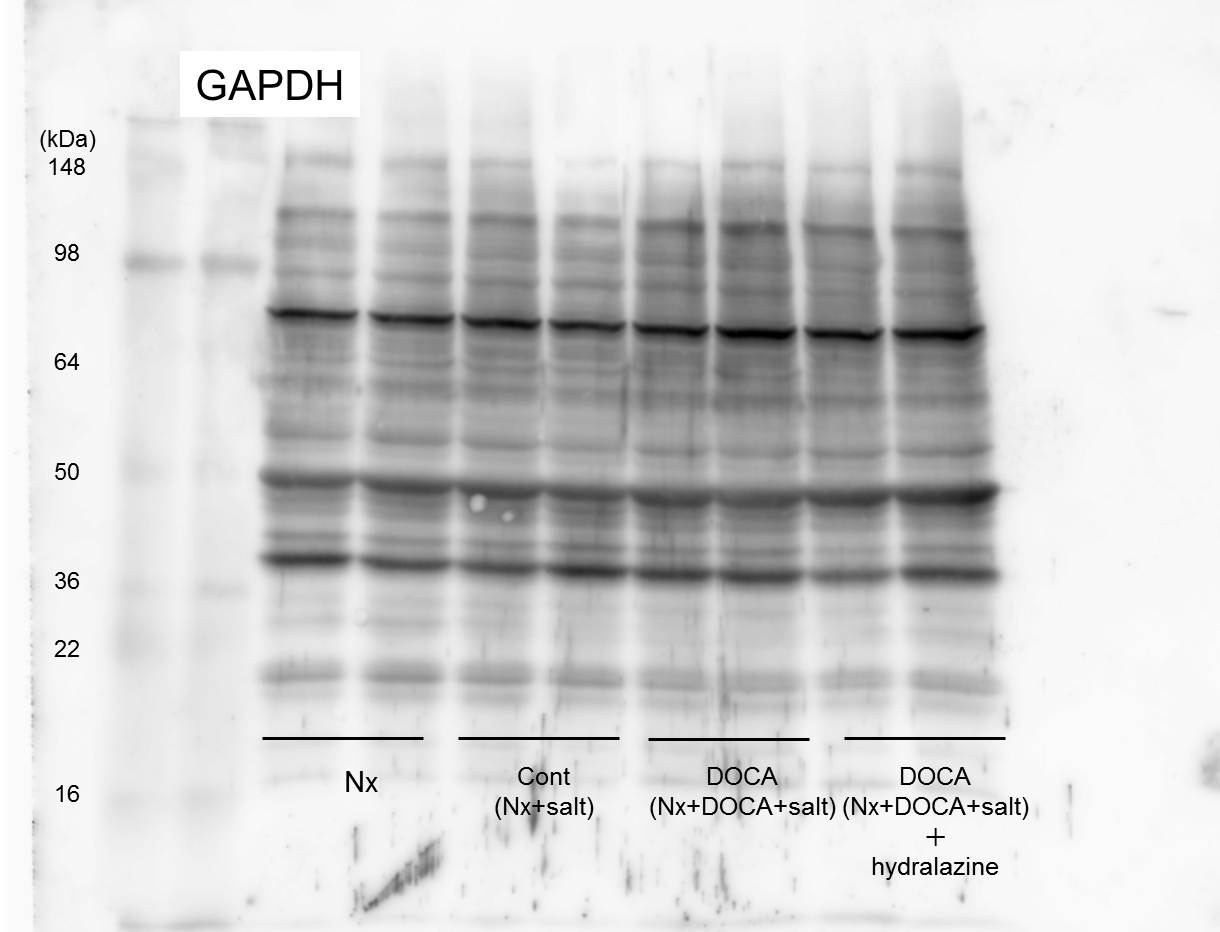
**

**
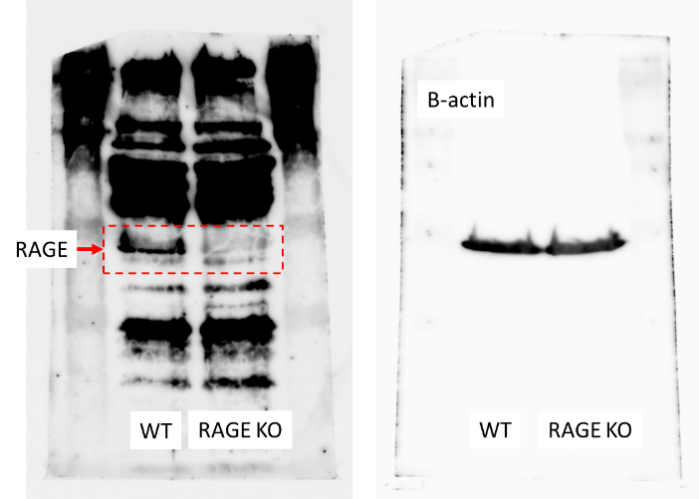
b**

**Supplementary Figure S6. Full length blots of Figure 2.**

Figure S5a and S5b showed the full length blots of Figure 2a and 2b, respectively.

Nx, uninephrectomy; salt, 4% salt diet: RAGE, receptor for advanced glycation end products; GAPDH, glyceraldehyde-3-phosphate dehydrogenase; WT, wild type. Red dotted lines show the cropping locations.

**Supplemen** **tary Figure S7**


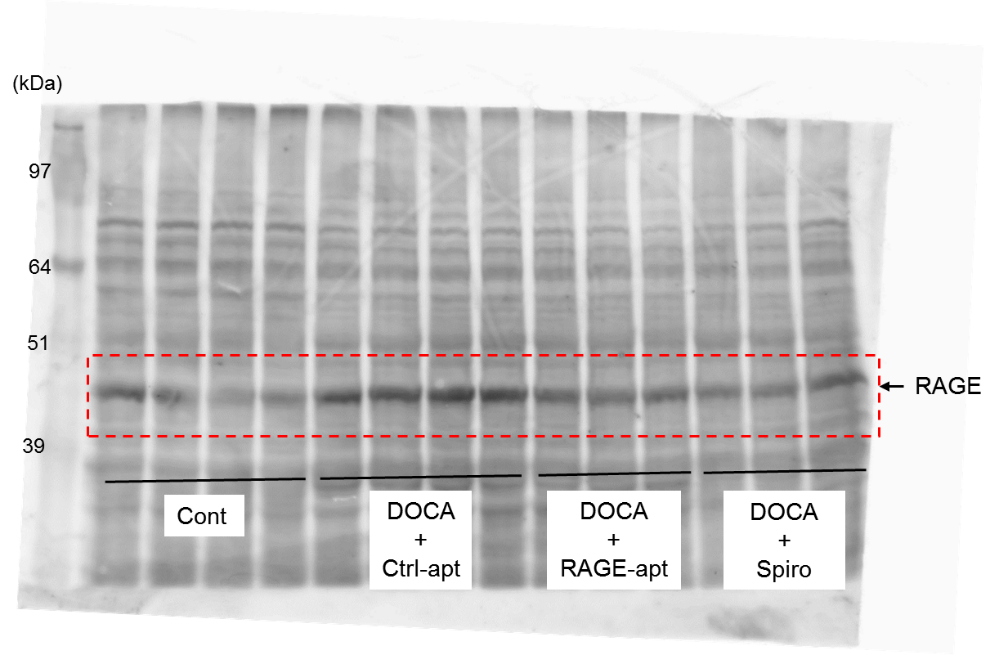


**
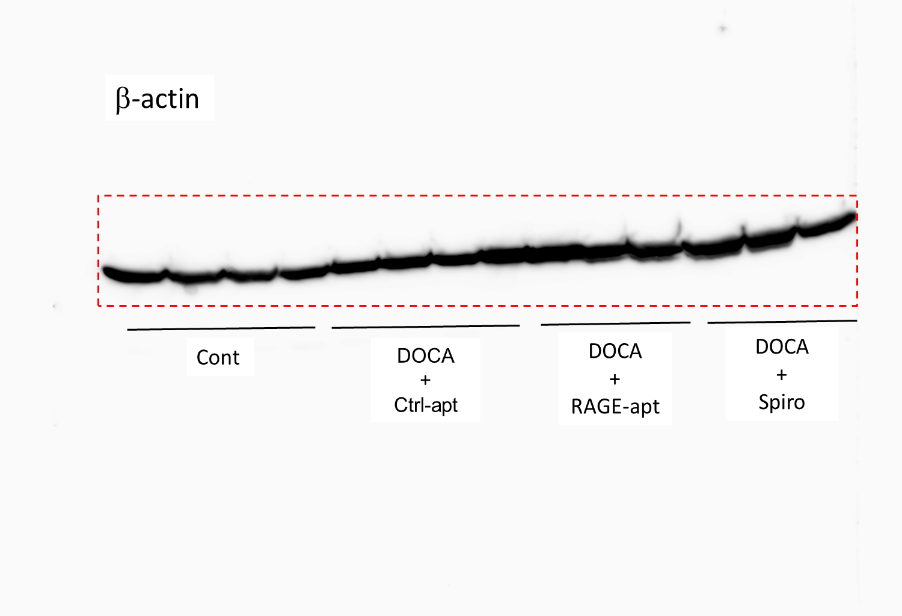
**

**Supplementary Figure S7. Full length blots of Figure 4i.**

RAGE, receptor for advanced glycation end products; Ctrl-apt, control aptamer; RAGE-apt, RAGE aptamer; Spiro, spironolactone. Red dotted lines show the cropping locations.

**Supplemen** **tary Figure S8**

**a**


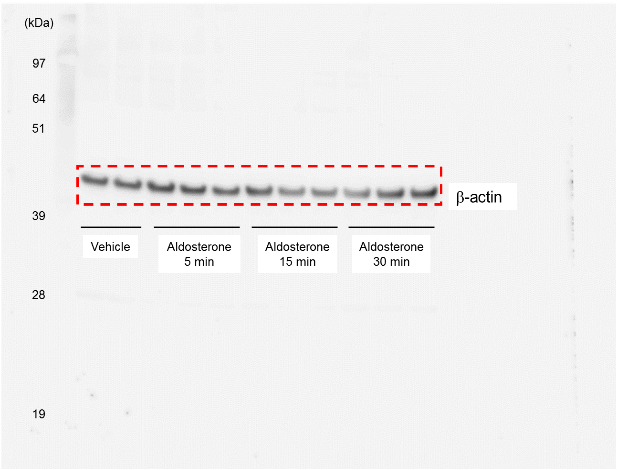

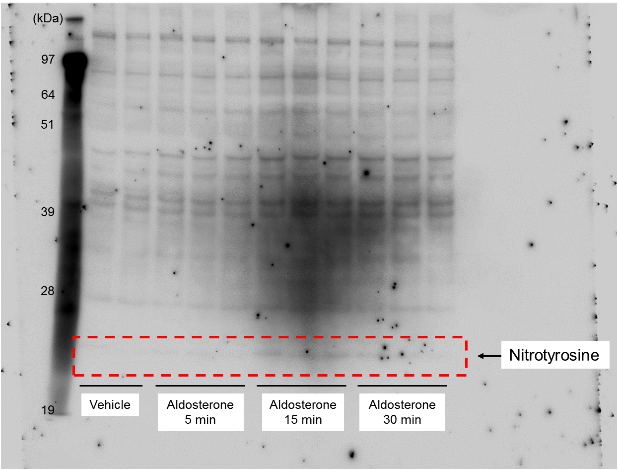


**b**


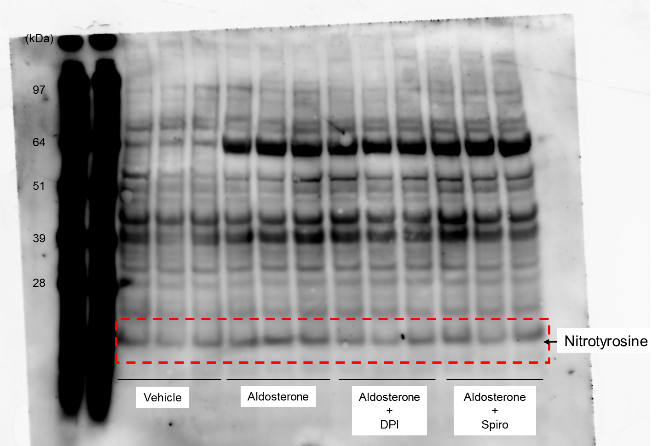


**
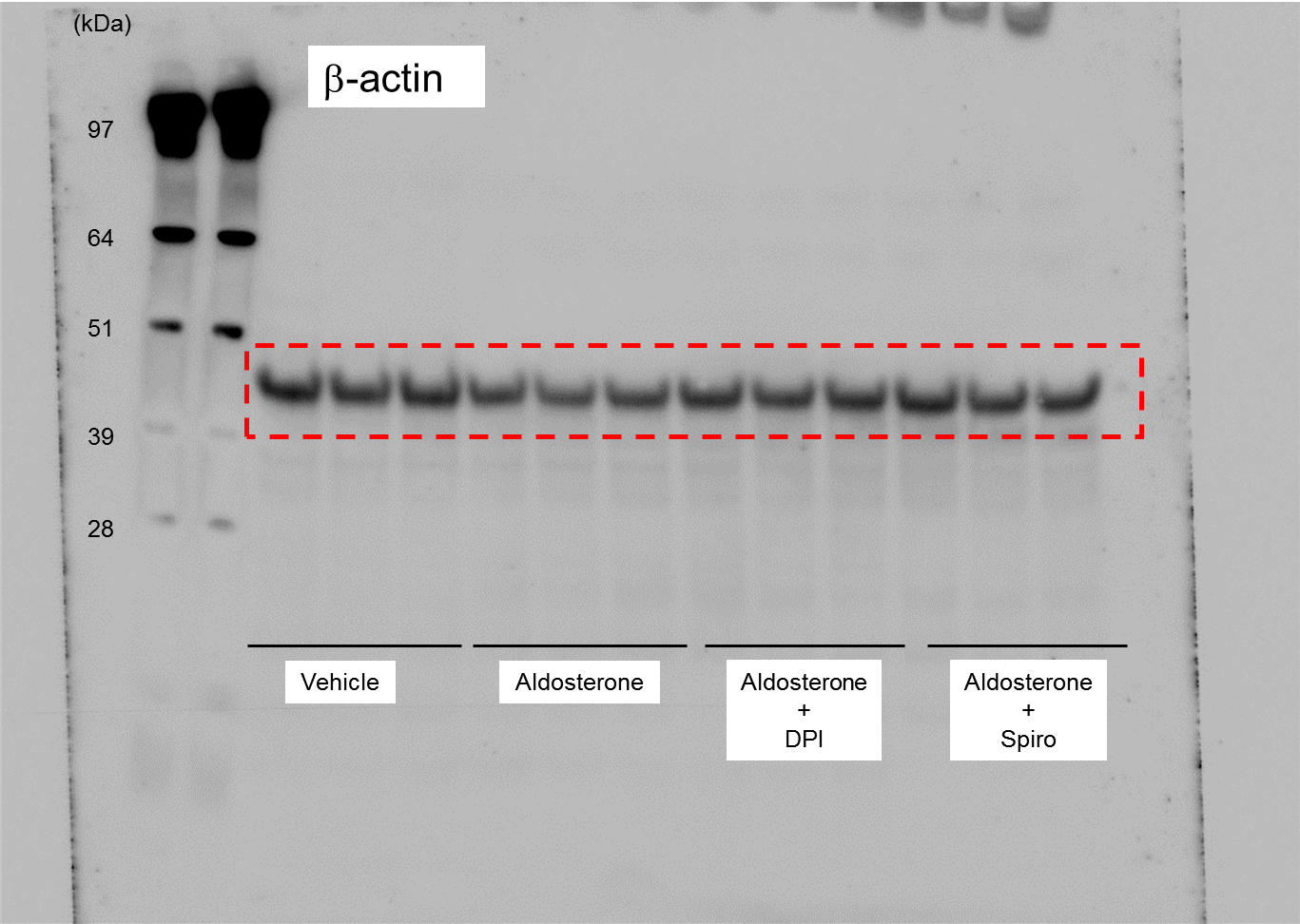
**


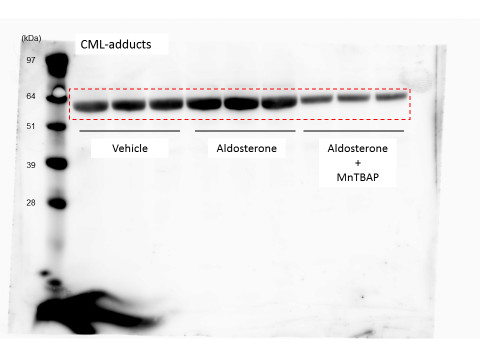
**c**


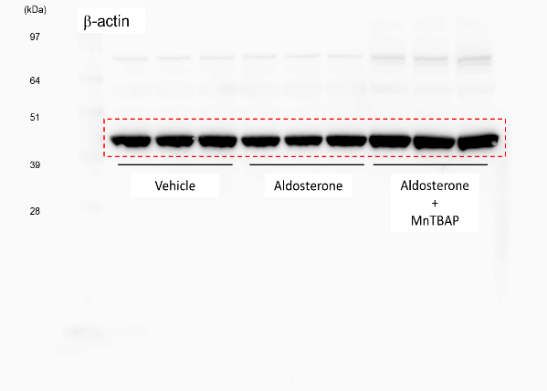


**Supplementary Figure S8. Full length blots of Figure 5.**

Figure S7a, S7b, and S7c showed the full length blots of Figure 5a, 5b, and 5d. The concentration of aldosterone was 1μM. DPI, Diphenyleneiodonium; Spiro, spironolactone: MnTBAP, manganese(III)tetrakis(4-benzoic acid)porphyrin. Red dotted lines show the cropping locations. Red dotted lines show the cropping locations.

**Supplemen** **tary Figure S9**

**a**


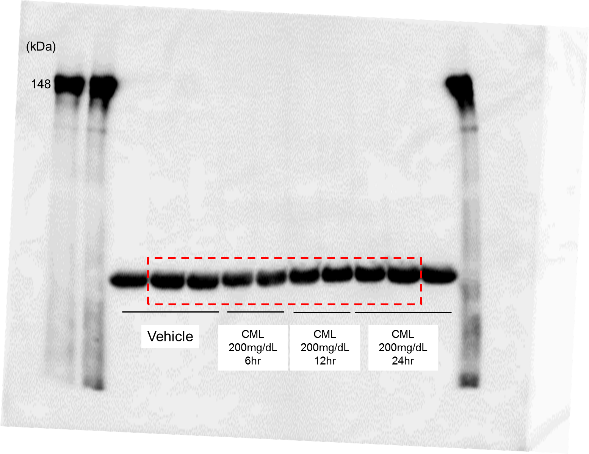
**
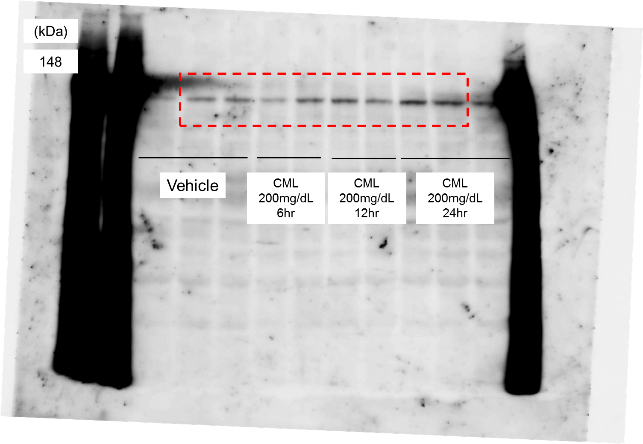
**

**
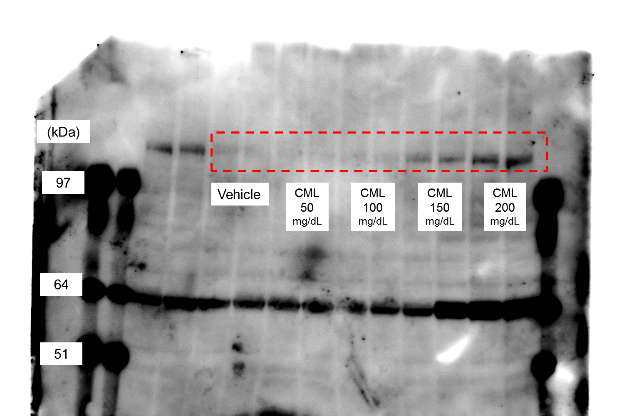
b**

**
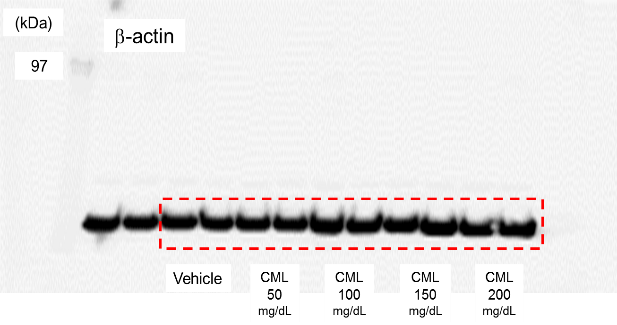
**

**c**


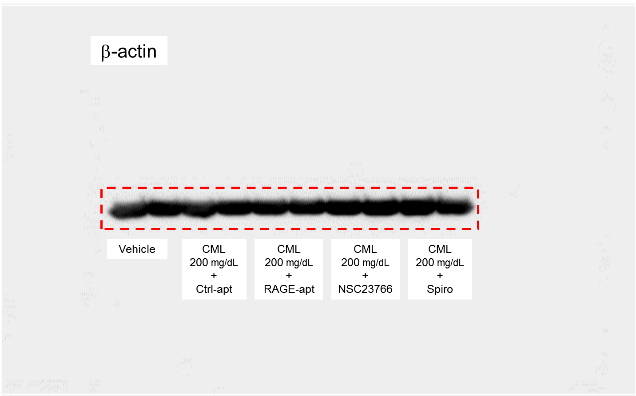
**
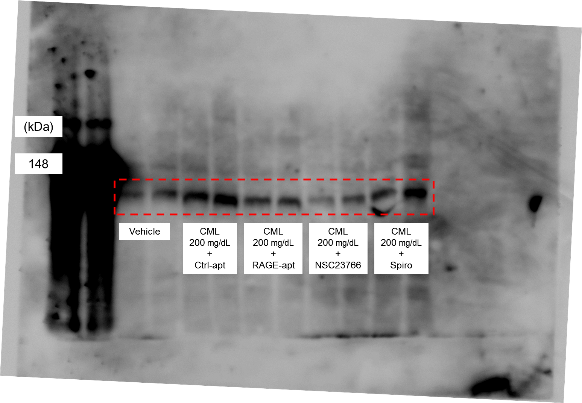
**

**Supplementary Figure S9. Full length blots of Figure 6.**

Figure S8a, S8b, and S8c showed the full length blots of Figure 6a, 6b, and 6c. Ctrl-apt, control aptamer; RAGE-apt, RAGE aptamer; NSC23766, Rac1 inhibitor; Spiro, spironolacton. Red dotted lines show the cropping locations.
